# Monitoring and Analysis of Ground Surface Settlement in Mining Clusters by SBAS-InSAR Technology

**DOI:** 10.3390/s22103711

**Published:** 2022-05-13

**Authors:** Huini Wang, Kanglun Li, Jun Zhang, Liang Hong, Hong Chi

**Affiliations:** 1 School of Civil Engineering and Architecture, Wuhan Institute of Technology, Wuhan 430074, China; wanghuini@wit.edu.cn (H.W.); 22004010017@stu.wit.edu.cn (K.L.); 22104010123@stu.wit.edu.cn (J.Z.); 2 Hubei Provincial Geographic National Conditions Monitoring Center, Wuhan 430070, China; hbchgis@126.com; 3 Key Laboratory for Environment and Disaster Monitoring and Evaluation of Hubei Province, Innovation Academy for Precision Measurement Science and Technology, Chinese Academy of Sciences, Wuhan 430077, China

**Keywords:** SBAS-InSAR, early identification method, mining area cluster area, surface subsidence

## Abstract

In this paper, we use the small baseline set technology and the early geological hazard identification method based on the selection of Permanent Scatter (PS) and Distributed Scatter (DS) points to carry out the research on surface deformation monitoring caused by underground activities in mining cluster areas. We adopted the Small Baseline Subset InSAR (SBAS-InSAR) technique to process Sentinel-1A SAR images over the research area from March 2017 to May 2021. The deformation estimation technology based on the robustness of PS points and DS points can be used for early identification of high-density surface subsidence in a large area of mines. The surface subsidence information can be obtained quickly and accurately, and the advantages of using InSAR technology to monitor long-time surface subsidence in complex mining cluster areas was explored in this study. By comparing the monitoring data of the Global Navigation Satellite System (GNSS) ground monitoring equipment, the accuracy error of large-scale surface settlement information is controlled within 8 mm, which has high accuracy. Meanwhile, according to the spatial characteristics of cluster mining areas, it is analyzed that the relationship between adjacent mining areas through groundwater easily leads to regional associated large-area settlement changes. Compared with the D-InSAR (Differential InSAR) technology applied in mine monitoring at the early stage, this proposed method can monitor a large range of long time series and optimize the problem of decoherence to some extent in mining cluster areas. It has important reference significance for early monitoring and early warning of subsidence disaster evolution in mining intensive areas.

## 1. Introduction

The large-scale exploitation of underground mineral resources will inevitably lead to the compression of underground rock formations [1,2], which will further contribute to surface subsidence [3] and slumping [4]. Surface subsidence and landslides not only destroy the original surface structure but also cause damage to surface buildings, groundwater pollution [2], and the destruction of arable land. The collapse of the surface of the mining area also impacts the safety of workers in narrow underground mines [5]. Therefore, it is important to explore methods for monitoring surface deformation in mining areas on a large scale with a long time series and a high accuracy.

The traditional subsidence monitoring technique mainly uses level surveys and the Global Navigation Satellite System (GNSS) to monitor the target area. Although it can obtain the change in the time series of subsidence, it has significant shortcomings: (1) the monitoring accuracy is affected by the quality of the GNSS receiver equipment [6]; (2) the reasonableness of the deployment of the GNSS points affects the accuracy of the deformation monitoring results [7]; (3) GNSS can only monitor single-point deformation information and so cannot accurately obtain polygon deformation data, while a study with a regional scope requires multi-point spatial decomposition to obtain regional results [7]; and (4) the traditional technique requires considerable human and material resources and has low spatial and temporal resolutions. Therefore, it is particularly ineffective when used for large-scale and long-time monitoring of a mining area, and it cannot capture all settlement changes in the mining area [8].

Interferometric Synthetic Aperture Radar (InSAR) has been widely used to monitor tailings dams [9], mine rehabilitation [10], building deformation [11], and mountain landslides [12,13,14,15] due to its all-day wide coverage, high accuracy, low cost, and low risk of acquisition data [16]. Since Carnec et al. [17] produced the first InSAR-based deformation map related to underground mining in 1996, significant progress has been made in the use of InSAR in monitoring mining deformation in the past 20 years. Mining-induced seismicity and associated surface deformation have been observed in the Upper Cilian coal basin with reference to seismological parameters, and the characteristics of the intense anthropogenic shaking caused by mining activities were investigated [18]. Due to the all-weather and high spatial coverage advantages of Differential InSAR (D-InSAR), Ou and Tan [19] used the dual-channel D-InSAR method to generate 19 pairs of interferometer images from 20 TerraSAR-X spotlight images, thus allowing the acquisition of high accuracy surface deformation data in the areas of the mine where no high-gradient deformation had occurred. However, the use of D-InSAR for long time-series surface deformation monitoring does not provide reliable subsidence information due to the atmospheric delay noise and the spatial and temporal decoherence [20]. This has led to the development of time-series-based InSAR techniques, of which Small Baseline Subset InSAR (SBAS-InSAR) overcomes the problem of decorrelation in areas of high deformation, and reduces the number of SAR images required for data processing [21]. To identify potential deformation hazards associated with rapid and uneven urbanization, Chen [22] used SBAS-InSAR to investigate the evolution characteristics of ground settlement and rebound in Xi’an during 2007–2019. He estimated that the ground settlement in Xi’an will generally decrease in the future and the rebound deformation area will continue to increase for a limited period.

Traditional SBAS-InSAR is unable to achieve reliable deformation monitoring results in the presence of large deformation gradients [23]. The presence of subsidence gradients and non-linearity in surface deformation caused by mining [24] is the combined product of groundwater extraction and structural changes in the mined area, and the deformation damage that occurs after mining is continuous [25]. However, limited by the side-view imaging configuration of SAR sensors [26], most studies have focused on the accurate measurement of 1-D or 2-D mining displacements for long periods along the radar line of sight (LOS) and/or in the azimuthal direction using SAR or InSAR techniques. Since mining deformation occurs in three dimensions [27] and is highly non-linear in both the spatial and temporal domains [28,29], it is difficult to identify the exact pattern of mining deformation from InSAR-derived 1-D or 2-D displacement observations. Singular value decomposition (SVD) methods are most commonly used to supplement deformation calculations in the vertical direction [30], which greatly limits the potential of using InSAR in mining deformation studies [15]. Due to the long history of coal mining in China and the disaster potential of deformation caused by internal structural damage in abandoned mining areas after deep mining, Donghui Chen and Huie Chen used the SBAS-InSAR technique based on 40 Sentinel-1A images for long-term subsidence monitoring to obtain time-series residual surface deformation information and analyze the effects of the underground coal mining and the potential mechanisms of stochastic subsidence associated with underground coal mining [20]. Therefore, it is important to provide early warning of potential threats and aid local mitigation based on the study of the evolutionary patterns of long-term subsidence and spatial deformation characteristics in mining areas.

In this study, the research object was the mining area in Daye City, Hubei, China, and the surface deformation within the area was monitored. Based on the Sentinel-1A data, the SBAS-InSAR technique was improved through adaptive filtering and other methods to obtain more accurate long time-series surface deformation monitoring results. Then, based on this, the deformation characteristics and development patterns of the mines in the research area were analyzed. GNSS data were used to present the results of the long time series surface deformation monitoring with higher accuracy.

## 2. Materials and Methods

### 2.1. Study Area

**Figure 1:**
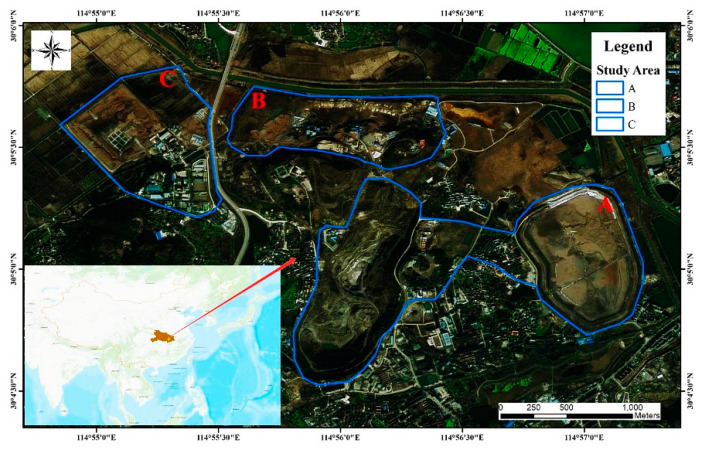
Location of the study area.

The study area is located in the southwest of Daye City, Hubei Province, China. The study area includes three mining areas: the Tonglv Mountain Mine (Area A), the Linihu Mine (Area B), and the Jiguanzui Mine (Area C) (Figure 1). The area has suffered varying degrees of damage to the mine surface environment due to the long history of continuous mining activities and underground mining, and it is a high-risk area for subsidence hazards. The study area is approximately 10 km^2^, with a maximum elevation of 20 m and a minimum elevation of 2 m. The lithology mainly consists of the Lower Triassic Daye Formation, the Lower Middle Triassic Jialing River Formation, the Lower Cretaceous Dashi Formation, and the Quaternary System, which are typical silica-type deposits. Most of the area is covered by the Quaternary deposits, and the carbonates of the Daye and Jialing River formations are closely related to copper–iron–gold mineralization. According to the analysis of the residual stratigraphy, the tectonics of the mining area are mainly folding and fracturing, and the folds in the study area are predominantly NWW and NNE trending. There are mainly three groups of fractures, with NW, NNE, and NE strikes. All of the fractures in the mining area have a small scale, and their lengths mostly do not exceed a few hundred meters [31]. The study area has been mined to a depth of up to 1065 m below the surface, and the mining method used was mainly post-void filling mining, supplemented by mechanized upward horizontal layered filling.

**Figure 2:**
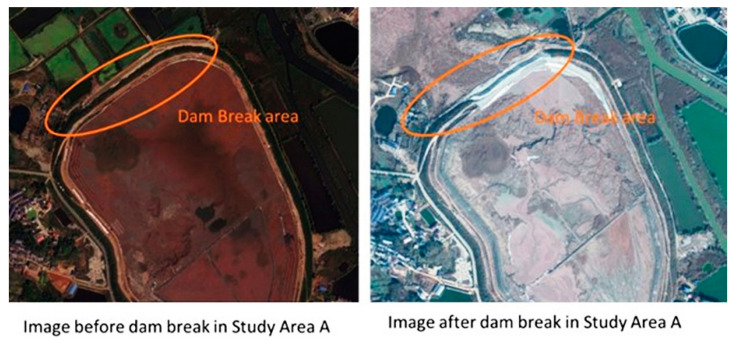
Images of the dam break area.

The copper and iron ore tailings reservoir in Study A are located on the north side of Shiquan Road, Quan Tang Village, Jinhu Street Office, Daye City (114°32′24″–114°54′54″ E, 30°4′33.6″–30°5′45.6″ N). It is a flatland-type tailings reservoir. Facing the water on the east, the reservoir is 1 km long, 0.7 km wide, elliptical in shape, and has a surface area of about 0.6 km^2^. The northwest section of the dam where an accident occurred is adjacent to dam copper and iron ore mine No. 3, and it is located 244 m, 172 m, and 108 m away from the old main shaft, the main shaft, and the dam failure accident, which was reported in the northwest dam section of the Tonglv Mountain. This failure resulted in two deaths and one missing person, with a direct economic loss of RMB 45,182,800. In recent years, the Ancient Copper Hill Copper Mine site has undergone several mine reversion and slope stabilization projects on the original basis. However, due to the excavation activities in the adjacent mine site, the perennial geological changes, and the natural subsidence effects, the mine site’s environment has become complex and diverse, including a decrease in the groundwater level caused by underground mining activities and the presence of loose soils and quicksand in some areas, all of which increase the potential for surface subsidence hazards. Images acquired before and after the dam break are shown in Figure 2.

This study combines the urgent need for dynamic monitoring of the mining geological environment in the region with SBAS-InSAR based surface deformation monitoring since the dam break occurred in March 2017.

### 2.2. Data

To comprehensively characterize the surface deformation in the study area, SAR Single-View Complex (SLC) images of the mining belt cluster in southwest Daye from 14 March 2017 to 16 May 2017 were selected, including a total of 124 views acquired in the C-band Sentinel-1A ascending orbit. The Digital Elevation Model (DEM) from the U.S. Geological Survey’s Shuttle Radar Topography Mission (SRTM) was used to simulate and remove the terrain phases. The data utilized for the calibration were provided by the Daye Geological Survey. The main parameters of the specific SLC data are presented in Table 1.

### 2.3. Methods

**Figure 3:**
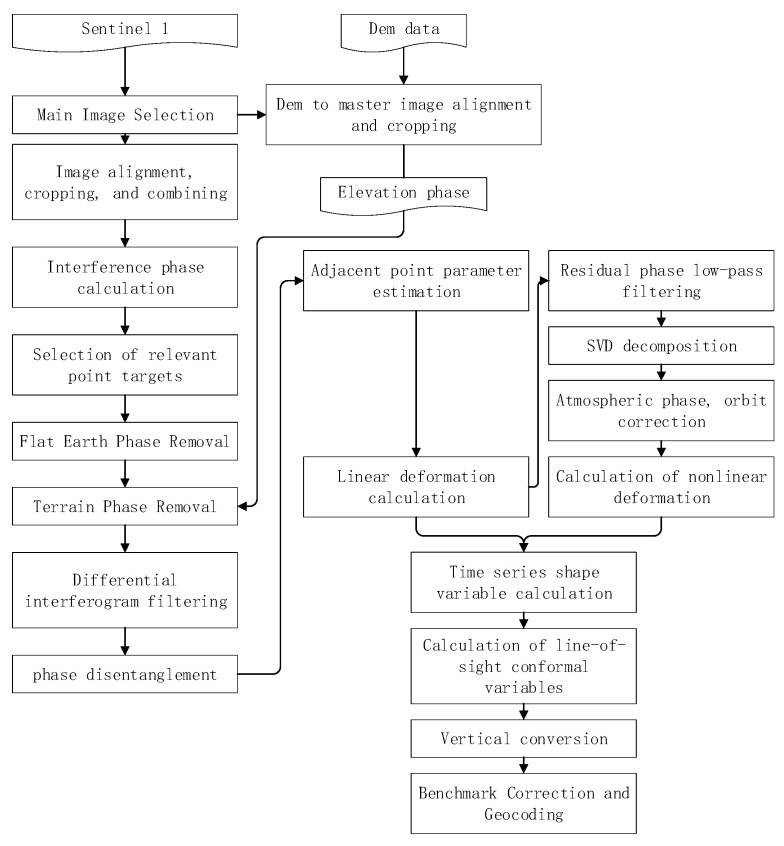
The flow chart of the study.

The data processing in this study was completed using the InSAR Scientific Computing Environment (ISCE) and Generic InSAR Analysis Toolbox (GIAnt) software for SBAS-InSAR regional time-series monitoring data processing, and the overall data processing workflow is shown in Figure 3.

Step 1: ISCE software is commonly used for interferometer processing, and a series of interferogram calculations can be completed based on the capabilities of this software prior to using the GIAnt software for temporal analysis. The database prepared included 124 views of SAR data and the original DEM of the study area.

**Figure 4:**
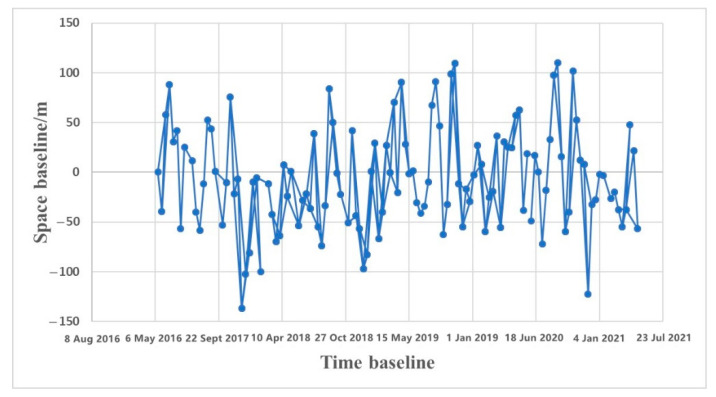
The information of space baseline and time baseline.

The SAR image acquired on 14 March 2017, i.e., the first view at the beginning of the time series monitoring, was selected as the main image. In this paper, the space baseline threshold is 200 m, and the time baseline is 24 d. All of the interferometer pairs that satisfied the time baseline were calculated, and then, the baseline spatial length of each pair was calculated, from which the relative spatial baseline lengths that satisfied the requirements were selected for the interferometer processing to complete the generation of the interferogram. The interferogram was obtained using 253 pairs. The interferometer pairs were screened according to their coherence and stripe quality, and the poor-quality pairs were removed. In total, 145 pairs were selected as final valid interferometer pairs. The spatiotemporal small baseline diagram obtained via differential interference is shown in Figure 4.

Since the quality of the interferograms generated by the interferometer pairs varied, the poor-quality interferograms needed to be screened out to improve the effectiveness of the timing calculations.

This completed the alignment of the master image, and the resulting coherent image was used as the selection image for the Permanent Scatter (PS) points, and the identification image was used for the Distributed Scatter (DS) points.

Step 2: The PS points were selected, and the temporal amplitude information was used to measure the phase noise level. In particular, in the high signal-to-noise ratio (SNR) image elements, the image element amplitude stability characteristics were used instead of the phase noise level to obtain the target point. For complex SAR images, the real part and imaginary part contain Gaussian noise with a standard deviation of $\sigma_{n}$:

(1) $$f_{\left(a\right)}=\frac{a}{\sigma_{n}^{2}}I_{0}\left(\frac{\mathrm{ag}}{\sigma_{n}^{2}}\right)e^{-\frac{a^{2}+g^{2}}{2\sigma_{n}^{2}}}\;\left(a>0\right)$$

where g is the energy reflected from the ground target; $\sigma_{n}\;$denotes standard deviation; a represents the pixel; $f_{\left(a\right)}$ represent amplitude; and I represents the imaginary part. The SNR is expressed as $g/\sigma_{n}^{2}$; when $g\gg \sigma_{n}^{2}$, the following relationship exists:

(2) $$\sigma_{A}\tilde{=}\sigma_{\mathrm{nR}}=\sigma_{\mathrm{nI}\;}$$

(3) $$\sigma_{v}\tilde{=}\frac{\sigma_{\mathrm{nI}}}{g}\tilde{=}\frac{\sigma_{A}}{m_{A}}=D_{A}$$

where $\sigma_{v}$ is the standard deviation of the phase, $\sigma_{A}\;$is the standard deviation of the temporal amplitude, $\sigma_{\mathrm{nR}\;}$ is the standard deviation of the real part of the complex, $\sigma_{\mathrm{nI}\;}$ is the standard deviation of the imaginary part of the complex, and $D_{A}$ is the amplitude deviation index.

The stability analysis of the detected PS points was carried out using the standard deviation of the temporal amplitude and the standard deviation of the phase.

Step 3: In the potential DS selection, the SAR images were calibrated, and then the calibrated SAR images were averaged to obtain the average amplitude map. The average amplitude map was used to identify the potential DS regions. The statistical homogeneity for each pixel point was determined using the Kolmogorov–Smirnov (KS) test, the relevant targets and persistent scattering targets were excluded using the classification information to obtain a rough DS candidate set, and then, the test was refined based on each of the candidate set’s statistical characteristics for the candidate objects for the refinement testing.

Step 4: The DS selection was optimized by taking the candidate set of the objects from the refinement test and using the Anderson–Darling (AD) visualization test. For pairs of pixels a and b, the AD statistic A² is

(4) $$A^{2}=\frac{N}{2}\underset{F_{a,b(x)}}{\sum }\frac{{\left[F_{a}\left(X\right)-F_{b}\left(X\right)\right]}^{2}}{\left[F_{a,b}\left(X\right)\times \left(1-F_{a,b}\left(X\right)\right)\right]}$$

where N is the number of SAR images, F_a_(X) and F_b_(X) are the empirical cumulative distribution functions of the amplitude X of pixels a and b, respectively, and F_a,b_(X) is the empirical joint cumulative distribution function of the combined pixel value of amplitude X. If the AD statistic value is less than the threshold value, then it is assumed that the two pixels belong to the same homogeneous region to obtain the adjacent points. When the candidate object is distributed in the same region with the adjacent points, the candidate object is selected, and the optimized selected DS point set is obtained.

Step 5: The interferogram processing of the SAR data was completed using the GIAnT software to create a time-series file from the coherence file and the interferogram in the above steps. A more stable area close to the urban area of Daye was selected as the reference area during the data processing. The coherence threshold was set to 0.4. The Delaunay Minimum Cost Flow (MCF) method for phase unwrapping was used, and the PS and DS were combined. The selected pixel points were connected to remove the atmospheric phase and to reduce the Atmospheric Phase Screen (APS) effect of the atmospheric phase. The selected measurement points were connected using the Delaunay triangulation network and the additional distance limitation principle. The triangulation network deformation parameters and the elevation correction values were estimated using the indirect parity observation method, and the typical trajectory and DEM error estimation were calculated using Equation (5).

(5) $$\Delta \varphi_{\mathrm{model}}^{k}=\frac{4\pi }{\lambda }\times T^{k}\times \Delta v_{\left(x,y\right)}+\frac{4\pi }{\lambda R\sin\theta }\times B_{⊥}^{k}\times \Delta \epsilon_{\left(x,\;y\right)}$$

where T^k^, $B_{⊥}^{k}$, R, and θ are the time baseline, normal baseline, tilt range, and angle of incidence, respectively, λ is the wavelength, and Δv and Δϵ are the deformation rate change and the elevation error change in the adjacent pixels, respectively.

Step 6: After completing the atmospheric and orbital error corrections, the configuration completed the calculation preparation file to complete the time-series deformation estimate, and the final deformation rate was calculated using Equation (6).

(6) $$\xi =\left|\frac{1}{N}\overset{N}{\underset{k=1}{\sum }}\exp\left[j\times \left(\Delta \emptyset_{<\mathrm{MML}:\;\mathrm{MROW}≯\mathrm{phase}}^{k}-\Delta \emptyset_{\mathrm{model}}^{k}\right)\right]\right|$$

where N is the number of interferograms, and k is the phase difference between two adjacent points in the kth channel interferogram. Based on the calculation of the time-series deformation variables, the original deformation variables were corrected through calculation of the line-of-sight direction (LOS), vertical deformation transformation, and geocoding. Finally, we obtained the long time-series baseline-corrected time-series map of the study area.

## 3. Results

### 3.1. Deformation Velocity

**Figure 5:**
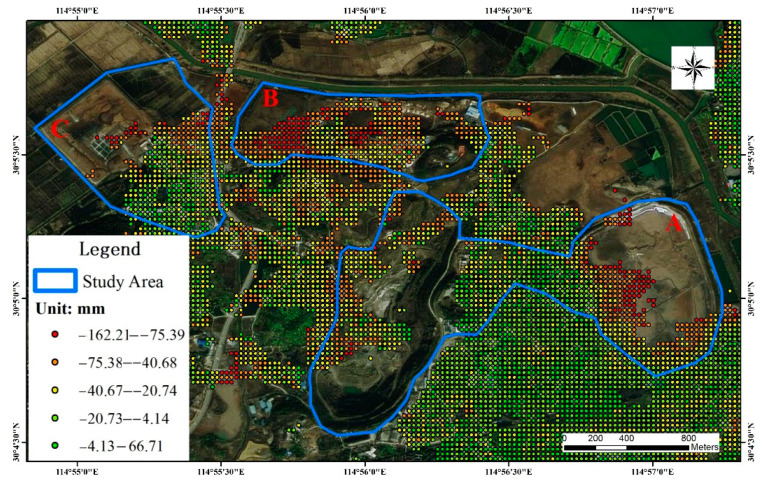
Regional settlement deformation data.

**Figure 6:**
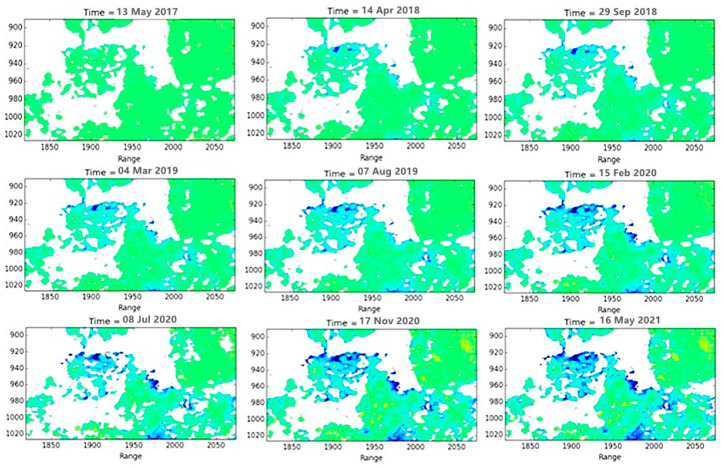
Map of time-series deformation processes.

The SBAS-InSAR time-series analysis method was used to obtain surface deformation rate maps for the period from 14 March 2017 to 16 May 2021 within the mining cluster area in Daye City. The results were superimposed on the sky map images, and the results were analyzed by superimposing the disaster point statistics (Figure 5). The positive values in Figure 6 indicate surface uplift in the vertical direction, and conversely, the negative values represent subsidence in the vertical direction.

During the study monitoring period, a large range of regional subsidence deformation occurred in the southern mining cluster area, and the most obvious deformation was observed in study areas A, B, and C. The locations where more geological hazards and subsidence deformation occurred were concentrated in the southern part of Daye City in the Tonglv Mountain mining cluster. Based on the field survey, it was determined that the mining history in this area is long, and the soil structure has become very unstable due to repeated excavation and back filling. In addition, the mountain in the area lacks vegetation cover due to the presence of open pit mining areas, making this area prone to landslides and other disasters induced by rainfall. Most of the mines in the area exist in clusters and have large slopes, which have caused multiple geological disaster situations during the long-term mining process, and there are multiple restoration and remediation engineering facilities in the area.

### 3.2. Time-Series Deformation

In this study, 124 views of the European Space Agency (ESA) Sentinel-1A satellite data from March 2017 to May 2021 were used to obtain the cumulative deformation variables at each time compared to the baseline data for 14 April 2017 using the SBAS-InSAR data processing method. The cumulative deformation data for the selected time points are shown in Figure 6. As can be seen from Figure 6, several major subsidence areas, including the southern Copper Hill mining cluster area (A, B, and C), gradually formed, and the cumulative subsidence and deformation impact areas gradually increased with time.

## 4. Discussion

### 4.1. Regional Subsidence

Field investigations and verification were carried out in the areas described as having large deformation, including separate field visits to study areas A, B, and C in the southwestern mining cluster area in Daye City.

(1) Study Area A

The Copper Hill mine is an ancient copper mining area that was mined historically, and its perennial excavation has left the geological environment of the mountain in a relatively unstable state. Due to the continuous excavation of the open pit, the mountain near the pit lacks vegetation cover and the soil is loose, making it prone to landslides and collapses induced by rainfall, which seriously threatens the lives and property of residents along the hillside. The presence of numerous underground robber holes distributed haphazardly has further damaged the underground soil structure of the area.

**Figure 7:**
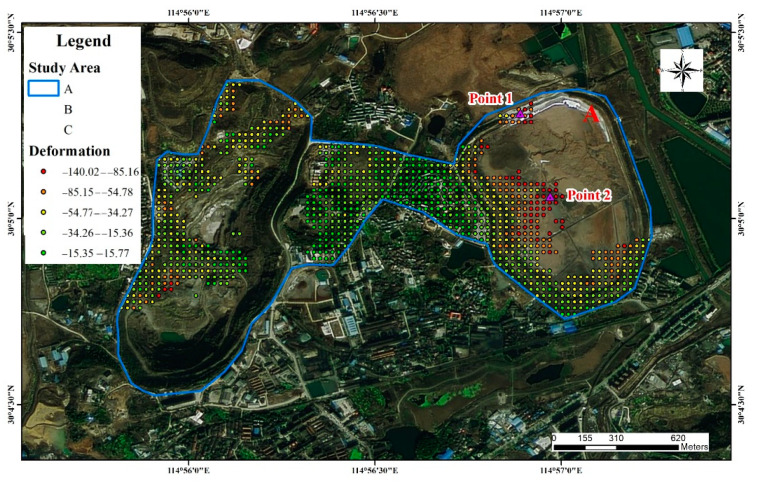
Site survey of study area A.

**Figure 8:**
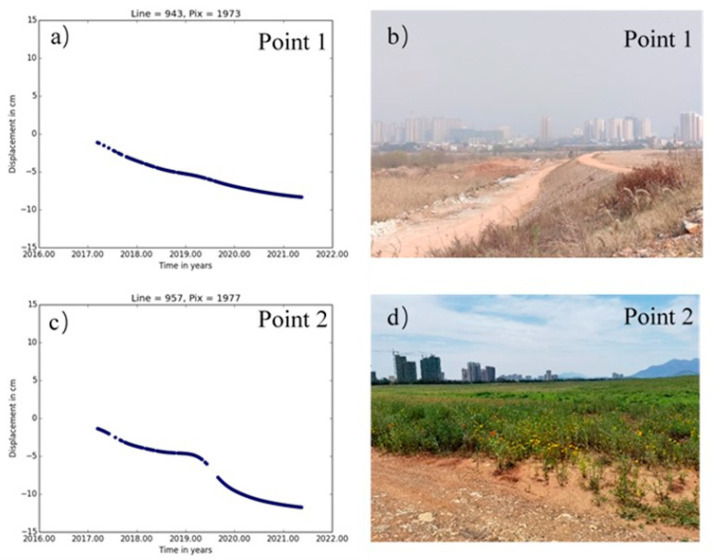
Time-series deformation and site survey for study area A, time-series deformation of Point 1 in (**a**), the photo of Point 1 in (**b**), time-series deformation for Point 2 in (**c**) and the photo of Point 2 in (**d**).

Figure 7 shows the settlement of study area A. Point 1 (114°56′53.287″ E, 30°5′16.734″ N) is located in the tailings pond dam break in area A. The time-series deformation process at point 1 from the field survey is shown in Figure 8a,b. Part of the deformation process is missing due to a large loss of coherence during the accident period in 2017, and the deformation trend at point 1 shows a gradual decline due to the natural settlement of the structure of the remediation building area after the tailings pond was repaired. The accumulated settlement at the end of the monitoring was −89.3 mm. The dam body at point 1 within study area A experienced a major dam failure on 20 March 2017, and the dam has been reinforced and repaired several times. The stability of the surface of the dam was affected by illegal mining and theft wells in private mining areas that existed under the previous dam’s body; this affected the accumulated subsidence of study area A during the monitoring period and the continuous subsidence of area A. The large settlement rate in area A may have been caused by two factors. First, there was a long time series of continuous subsidence after the repair. Second, there is still a mining area below the area, which has resulted in continuous subsidence. Figure 8c,d show the time-series deformation process and field survey at point 2, respectively. Point 2 was originally a reservoir on the dam body. After filling and repair of the reservoir in 2017, the newly filled soil was loose and prone to natural settlement induced by rainfall, which is the main reason for the large deformation and settlement in this area. The cumulative deformation at the end of the monitoring period was −136.6 mm.

(2) Study area B

**Figure 9:**
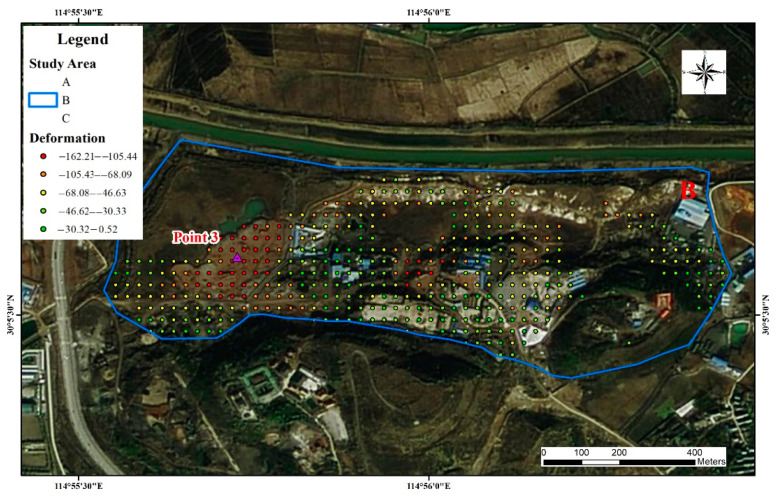
Site survey of study area B.

**Figure 10:**
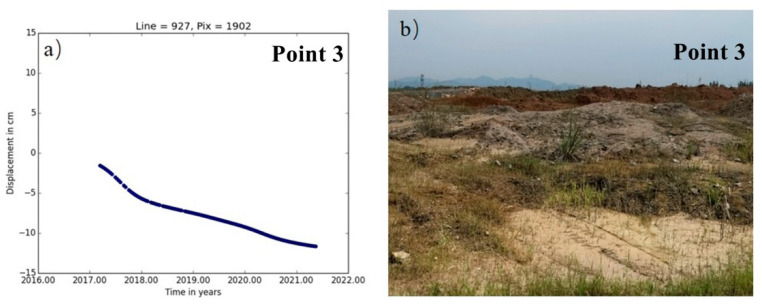
Time-series deformation and site survey of study area B, time-series deformation of Point 3 (**a**), the photo of Point 3 (**b**).

The mine in this study area is a medium-sized copper and iron ore deposit, though it is mainly copper. Located in the Daye Lake Basin area, it has a well-developed surface water system, blurred geological boundaries, karst-filled deposits, and complex geological conditions in the mining area in Figure 9. The main water-filled surrounding rocks are composed of fissure cave aquifers, which are karst-developed and water-rich. The lithology of the strata in the mining area is complex and fracture structures are developed. The main environmental geological problems that exist in the mine are karst ground subsidence induced by mining pit drainage and surface water backing up along the collapsed pit. The time-series deformation process and field survey at point 3 are shown in Figure 10a,b. In the strip of subsidence deformation, the average subsidence rate was −12 mm/a to −26 mm/a. Point 3 is located in the abandoned mining area in study area B. There is a large number of abandoned underground mining areas at point 3, and the upper part of the surface consists of sediment-type accumulation soil that easily slides. The accumulated settlement at the end of the monitoring period was −143.3 mm.

(3) Study area C

**Figure 11:**
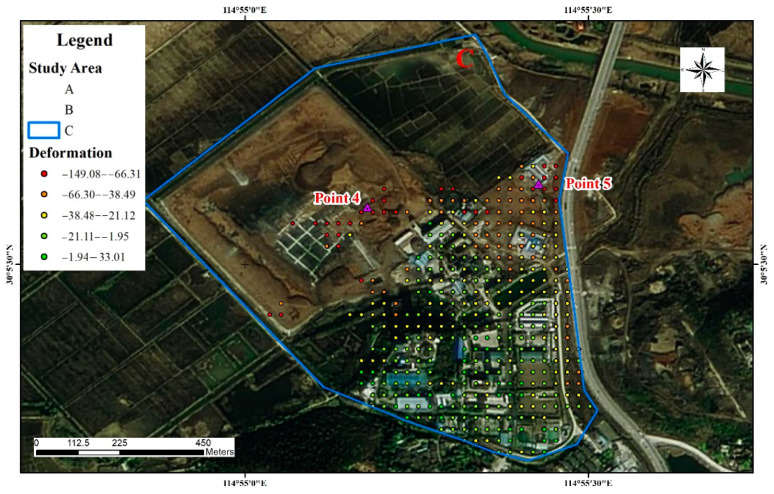
Site survey of study area C.

**Figure 12:**
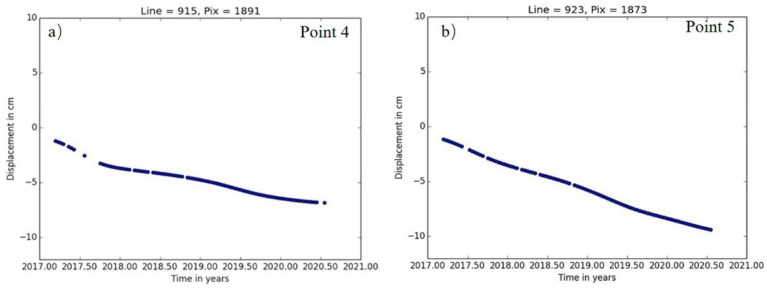
Time-series deformation in study area C, time-series deformation of Point 4 (**a**), the photo of Point 4 (**b**).

The main deformation areas in the mine area of the Hubei Sanxin Gold and Copper Company, Limited are shown in Figure 11. The working area, the tailings pond, and the underground mining area all exhibit varying degrees of deformation, and the largest cumulative deformation settlement is concentrated in the northern part of the settlement area. Points 4 and 5 are located in the mining area in study area C. This is consistent with the trend of natural settlement and is not a deformation factor constituting a geo-environmental hazard. The deformation time sequence diagram is shown in Figure 11, but some deformation also occurred in parts of the working area and underground extraction area, and although the mining activities at this location mainly use the back filling while mining method, the impact of the damage to the soil structure already caused is still irreversible. Figure 12a shows the time-series deformation process at point 4. Point 4 is located in the road facilities that are affected by the mine and produce ground settlement. The accumulated settlement was −61.4 mm.

There are several deep mining areas underneath this point that are still being mined, and there is an interconnection between the groundwater and study area B. Figure 12b shows the time-series deformation process at point 5. The change in the trend during the monitoring period was similar to that at point 3, and the cumulative settlement was −106.5 mm at point 5.

**Figure 13:**
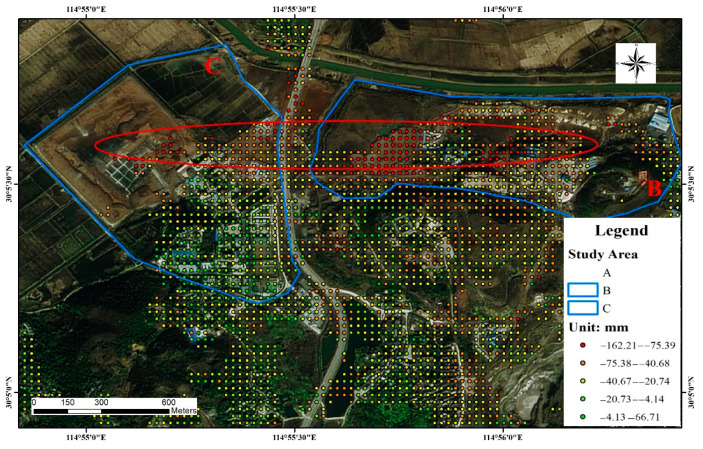
Study area B and C time-series deformation and site survey.

The underground mined-out areas in study areas B and C (red elliptic area in Figure 13) were connected by groundwater and affected by the adjacent Daye Lake under the influence of mining activities and banded and large-scale surface subsidence. Tongdu Avenue, located between the two mining areas, also exhibited deformation with the obvious displacement of a platform near the Changliugang Bridge.

### 4.2. Validation with GNSS

**Figure 14:**
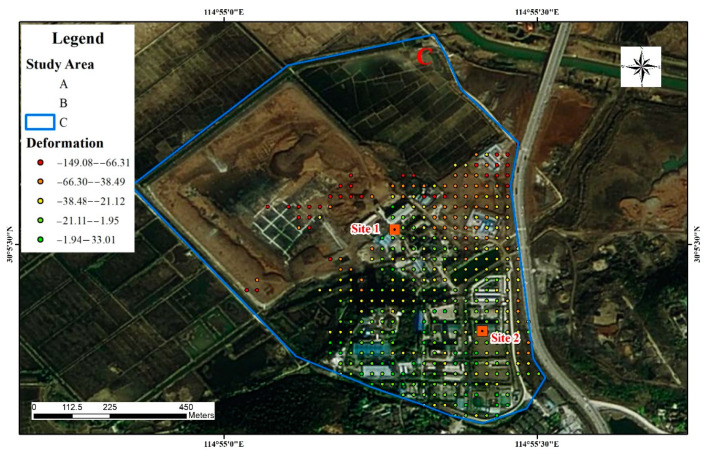
Monitoring point layout in study area C.

To further corroborate the accuracy of the results derived from the SBAS-InSAR processing, GNSS level monitoring data based on the field mineral rights company in the key monitoring area were compared to the results. Two level monitoring points (site 1 and site 2) were set within the study area where subsidence was detected at the Cockscomb Mine. The specific monitoring locations are denoted by the yellow rectangles in Figure 14. The monitoring period for the level surveys was from 16 July 2017 to 20 July 2020.

The cumulative deformation extracted using the SBAS-InSAR technique was used to produce a deformation time-series diagram for visual comparison of the monitoring results with temporal deformation monitored by the GNSS method.

**Figure 15:**
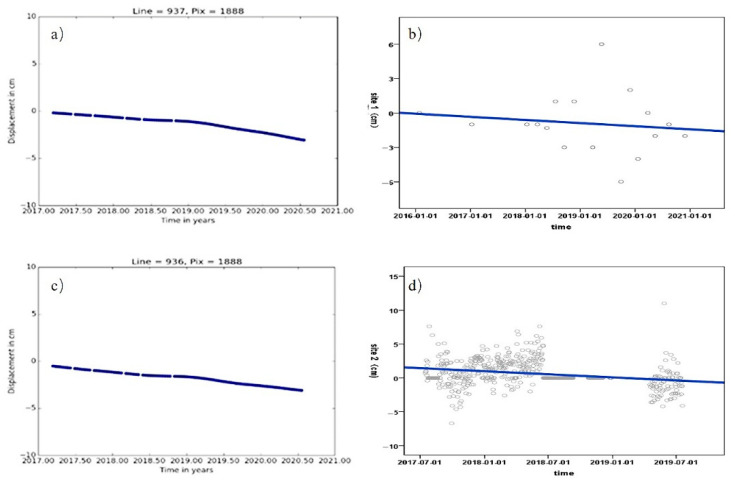
Deformation trend level calibration chart, time-series deformation of Site 1 in (**a**), GNSS time series observation data trends of Site 1 in (**b**), time-series deformation of Site 2 in (**c**) and GNSS time series observation data trends of Site 2 in (**d**).

Figure 15a,b show the site 1 time-series deformation process and the corresponding monitoring data fitting trend (linear fitting), and Figure 15c,d show the site 2 time-series deformation process and the corresponding monitoring data fitting trend (linear fitting). As can be seen from the legend, the measured cumulative settlement at GNSS sites 1 and 2 during the level observation dates were −1 mm and −3 mm, respectively, while the SBAS-InSAR monitored cumulative settlement at sites 1 and 2 were −2.63 mm and −1.2 mm, respectively. The errors in the monitoring results were −1.63 mm and −1.8 mm respectively, i.e., both were less than 5 mm, which quantitatively demonstrates the accuracy of the SBAS-InSAR.

## 5. Conclusions

Based on radar image data from the C-band Sentinel-1A uplift, in this study, the SBAS-InSAR technique was used to monitor the subsidence in the southwestern mining cluster in Daye Lake, Daye City, Hubei Province. The cumulative surface subsidence and time-series curves for the study area were analyzed, and the monitoring results were corroborated through a comparison of the site survey and historical data. The results show that continuous surface settlement changes occurred in the study area between 14 March 2017 and 16 May 2021, and the subsidence was mainly concentrated in the tailings pond dams, the slopes of the open pit mines, and the surfaces above underground mining areas.

In addition, compared with GNSS monitoring data of the same period, the deformation trend and accuracy of the method used in this work are in good agreement. Our findings suggested that the SBAS-InSAR method is well suited to monitor subsidence on a large scale and over a long period. The monitoring results matched the actual locations and extents of subsidence and were consistent with the historical mining progress, thus allowing for the monitoring of the identified hazards and the identification of hidden subsidence hazards. Therefore, the proposed method could be an important tool in monitoring potential subsidence hazards during mining and implementing timely preventative strategies.

## Figures and Tables

**Table 1:** The description of SAR parameter.

Parameter	Value
**Track**	17
**Beam mode**	IW Mode
**Angle/(°)**	36.7
**Polarization**	VV
**Flight direction**	Ascending
**Number of images**	124
**Start time**	14 March 2017
**Stop time**	16 May 2021

## Data Availability

Not applicable.
